# From Cow to Climate—Tracing the Path of Dairy Sustainability: Unveiling the Impact on Sustainable Development Goals Through Bibliometric and Literature Analyses

**DOI:** 10.3390/ani15070931

**Published:** 2025-03-24

**Authors:** Douglas Mwirigi, Mária Fekete-Farkas, Csaba Borbély

**Affiliations:** 1 Doctoral School of Economics and Regional Sciences, Hungarian University of Agriculture and Life Sciences, Páter Károly u. 1, 2100 Gödöllő, Hungary; 2 Institute of Agricultural and Food Economics, Hungarian University of Agriculture and Life Sciences, Páter Károly u. 1, 2100 Gödöllő, Hungary; farkasne.fekete.maria@uni-mate.hu; 3 Institute of Agricultural Economics and Food Economics, Hungarian University of Agriculture and Life Sciences, Páter Károly u. 1, 2100 Gödöllő, Hungary; borbely.csaba@uni-mate.hu

**Keywords:** bibliometric analysis, climate, cow, dairy farming, environmental impact, milk production, sustainability, sustainable development goals, sustainable practices

## Abstract

Archeological evidence shows that dairy farming dates to the early Neolithic era in Europe, the Middle East, Asia, and Africa. Over time, it has evolved from domestication to intensive dairy farms with large, high-tech processing units. Dairy farming has contributed to economic growth, food production, employment, and processing industries. Nonetheless, it has been identified as a major contributor to climate change. This study explores the literature on dairy farming and sustainable development goals (SDGs) to identify current scholarly developments since the formulation and adoption of the SDGs in 2015 and themes for future research. This paper argues that sustainability shortfalls in dairy farming are primarily driven by human processes associated with commercialization and industrialization rather than the animals themselves, although biological emissions remain an inherent factor. Data were analyzed using R package, Excel, NVIVO, and VoS Viewer. A review of the literature showed that dairy farming and its contribution to sustainability has gained more scientific interest since 2015. Moreover, livestock management, feed production and management, stakeholder management, logistics and supply chain management, and waste management are the sources of environmental adversities associated with dairy farming. Notably, these are human processes developed from the commercialization of dairy farming and involve multiple stakeholders across the supply chain. While solutions are embedded within these processes, innovation emerges as a key driver of sustainability and a source of opportunities to strengthen sustainability in the dairy farming sector and achieve SDGs. Sustainability strategies, such as sustainable intensification, multifunctional agriculture, and agro-ecology should be implemented to improve sustainability in the dairy sector.

## Simple Summary

The dairy industry is an important agricultural sector in the global economy that supplies food, offers job opportunities, and supports industries. Nonetheless, the sector’s contribution to climate change has drawn attention from scholars, policy formulators, and industries as they grapple with developing approaches to mitigate global warming. This study explores the literature on dairy farming and sustainable development goals (SDGs) to identify current scholarly developments since the adoption of the SDGs in 2015 and highlights themes for future research. The study established that the contribution of dairy farming to sustainability has gained interest since 2015. Moreover, dairy sustainability research is evolving, and priorities are shifting from traditional production concerns to holistic environmental management approaches. Notably, human processes, such as livestock management, feed production and management, stakeholder management, logistics and supply chain management, and waste management, are the major sources of environmental adversities associated with dairy farming. This study recommends adopting innovative technologies and sustainable management systems to address emerging sustainability challenges in the sector.

## 1. Introduction

Dairy farming is a source of revenue, food, and employment to the global economy. More specifically, it benefits the world by providing milk and other dairy products [1] However, despite the multiple benefits associated with this industry, it has been implored for its potential adverse impacts on the environment [2]. While dairy farming has existed for centuries, increased greenhouse gas emissions and intensified production has made it become a global concern in the past 80 years. The main contributions of the sector to sustainability challenges are greenhouse gas emissions, water pollution, soil degradation, and changes in land use. Pollution in this context is a product of imbalance attributed to excess nitrogen and carbon in locations where they cannot be recycled effectively. This section begins by exploring the shortcomings associated with dairy farming in relation to sustainable development to develop an understanding of how the sector affects the environment. The subsequent section explores the strategies proposed by numerous studies to address sustainability shortcomings.

The dairy sector has been identified as a major source of greenhouse gas emissions through waste and enteric fermentation [3]. Dairy cattle excrete nitrogen emissions through their urine and fecal matter [4]. The United Nations (UN) Food and Agriculture Organization estimates that the sector accounts for 4.9% of the total human-induced emissions [5]. Additionally, processing dairy products pollutes water and air and contributes heavily to deforestation and soil degradation [6].

The past thirty years have been characterized by global warming. This period has been the warmest in the history of the earth [7]. Besides the surge in temperature, the world has also experienced other major changes to the climate, such as rising sea levels, increasing ocean temperatures, extreme weather events and related disasters, and substantial rises in the emission of greenhouse gases [8]. The past few years have been marked by increases in the oceanic uptake of carbon, resulting in ocean acidification and a reduction in the surface water pH [9]. These changes primarily result from anthropogenic emissions of greenhouse gases that have steadily risen since the commencement of the industrial revolution in the 1750s [10]. Livestock farming, including the phases of growing fodder crops, processing and transporting animal products, and consumption, has a relatively significant impact on climate change [11].

Predominantly, dairy farming has been demonstrated to adversely impact land, water, and air. In terms of air quality, the dairy industry accounts for 2.9% of global emissions, which is a significant percentage [12]. Apart from carbon emissions, it contributes to other forms of air pollution, including ammonia emissions [4]. The enteric fermentation and manure processes also compromise air quality by producing methane [13]. As potent greenhouse gases, carbon, ammonia, and methane are highly effective in wrapping heat in the atmosphere, a core factor contributing to global warming [8]. These emissions also contribute to the development of particulate matter and secondary aerosols, which adversely affect the environment and human health [14].

The adverse impacts of dairy farming processes also extend to water quality and land degradation. The processes involved in dairy farming affect the quality of water by discharging pathogens, nutrients, and other contaminants into water bodies [12]. Discharge occurs in the form of runoff from dairy cows. Besides contaminating water, runoff causes eutrophication by releasing nutrients like phosphorus and nitrogen into nearby waterways. Eutrophication causes algal blooms that result in the depletion of oxygen levels in water bodies [12]. In addition to killing fish and other marine animals, eutrophication compromises the entire aquatic ecosystem. Intense dairy farming practices are often linked to land degradation issues due to the overuse of synthetic fertilizers and manure [13]. This often results in soil compaction, erosion, and nutrient imbalances [15]. These adverse effects have caused intense pressure to embrace sustainable practices to not only mitigate them but also foster ecological stewardship in addressing the global warming issue.

There are multiple strategies that numerous studies have proposed to address sustainability issues in dairy farming. They include sustainable intensification, multifunctional agriculture, and agroecology. Each of these strategies is explained in Section 1.1, Section 1.2, and Section 1.3, respectively.

### 1.1. Sustainable Intensification

One recommended tactic for eliminating the shortcomings and mitigating the dairy sector’s adverse impact on the environment is sustainable intensification (SI). SI is a strategy that acknowledges the need to intensify food production to satisfy the expanding demand [16]. It recognizes the negative ecological impacts of agricultural production on the environment and seeks to increase food production without encroaching on both resources and the environment by highlighting the significance of expanding productivity per unit land. SI strategies in dairy farming target ecological issues, such as minimizing greenhouse emissions by breeding cows in a manner that reduces the emission of methane and planting feeds that require less nitrogen fertilizers [17].

This strategy resonates with the SDGs of zero hunger, clean water and sanitation, climate action, and responsible consumption and production. SDG 2, zero hunger, seeks to improve accessibility to food for the global population. Therefore, by utilizing sustainable practices like optimized feed efficiency to improve the productivity of dairy cattle, SI contributes to the second SDG [18]. It achieves this by improving food production and safeguarding food security. For clean water and sanitation (SDG 6) and climate action (SDG 13), SI fosters responsible water use, minimizes water pollution, curtails the emission of greenhouse gases, and increases carbon sequestration as asserted [17]. This strategy aligns with the core objectives of responsible consumption and production (SDG 12) as it fosters resource optimization and waste minimization. Dairy farmers align with the principles of responsible production and consumption patterns by utilizing SI practices to reduce food waste and improve productivity. Nonetheless, there exist multiple barriers to the implementation of sustainable intensification [19]. Socio-economic barriers, such as financial constraints, market access, and knowledge training, and technological barriers, such as unavailability of appropriate technology in underdeveloped areas and the complexity of sustainable intensification practices like conservation agriculture, deter farmers from adopting them [20].

### 1.2. Multifunctional Agriculture

Besides SI, the other development that has emerged in this sector to foster ecological sustainability efforts is multifunctional agriculture. It is a non-productivism paradigm that restructures the rural landscape to allow diverse services beyond agricultural production [21]. This strategy usually reintegrates agriculture with rural development initiatives, such as poverty reduction, and merges them with environmental conservation. The approach fosters the shift from a sectoral focus on agriculture to a more holistic emphasis on regions [22]. It enables policymakers to address human–environmental elements of multifunctional landscapes, such as the diversification of livelihoods. Thus, it best resonates with the first SDG, which seeks to eliminate poverty, SDG 10 (reduced inequalities), and SDG 5 (gender equality).

In relation to SDG 1 (no poverty), the multifunctional agriculture strategy contributes to the eradication of poverty by providing farmers, especially small-scale farmers, with the opportunity to venture into diverse livelihoods and income sources. It achieves this by incorporating multiple functions, such as rural development, food production, and ecosystem services [23]. The 10th SDG (Reduced Inequalities) relates to this strategy by fostering more equitable and inclusive rural development. It supports smallholder farmers and marginalized groups like women and indigenous communities by increasing their access to opportunities and resources [22]. Therefore, by supporting women, multifunctional agriculture also aligns with the gender equality goal (SDG 5). It empowers women to participate in agriculture.

### 1.3. Agro-Ecology

Agro-ecology is a development that fosters ecological sustainability in the dairy sector. This strategy dates to the 1930s, and it can be defined both as a scientific approach and a social movement/practice [16]. It fuses ecology and agronomy to develop a sustainable food system. As both a social movement and a scientific discipline, agro-ecology accentuates the integration of social and natural science, practical engagement with activist groups, and close interactions with farmers to catalyze social change [24]. It is widely recognized as the most appropriate strategy to address the unbalanced power dynamics that inform dairy intensification trends [25]. Therefore, it resonates with industry, innovation, and infrastructure (SDG 9), reduced inequalities (SDG 10), life below water (SDG 14), and life on land (SDG 15).

Agro-ecology aligns with the core aspects of SDG 9 by fostering innovation in the dairy sector in the form of sustainable farming practices. In reducing inequalities (SDG 10), the practice promotes more equitable and inclusive farming practices. It achieves this by empowering smallholder farmers and marginalized communities and increasing their access to resources [26]. Therefore, it minimizes agricultural productivity and income disparities [16]. SDGs 14 and 15 address environmental pollution directly since they advocate for the conservation of both marine and freshwater ecosystems and biodiversity. Agro-ecology not only promotes practices that reduce the use of agrochemicals but also promotes farming practices like agroforestry [24]. Therefore, the practice helps to preserve ecosystem and aquatic biodiversity by reducing water pollution, contamination, and agricultural runoff [27]. Nonetheless, there exist multiple barriers to the adoption of agro-ecology due to variations in economic, social, and political aspects across the globe [28]. The high cost of transitioning to agro-ecological practices, a lack of developed markets for agro-ecological products, lack of awareness and understanding of agroecological practices, cultural resistance, weak policy frameworks, conflicting political and economic interests, and a shortage of skilled advisors and technical support limit farmers’ ability to adopt and maintain agro-ecological practices [29].

Despite the increased focus on sustainable agricultural practices, there has been limited consolidated knowledge on the key contributors, publication trends, and thematic changes in sustainability and dairy farming research over the past decade. The purpose of this research is to explore the literature on dairy farming and SDGs to identify current scholarly developments since the formulation and adoption of the SDGs in 2015 and themes for future research. To explore current developments in dairy farming, this study seeks to address the following research questions.

RQ1. What were the publication trends in dairy farming and sustainability from 2015 to 2023?

RQ2. Who were the most prolific scholars, articles, journals, and countries contributing to dairy farming and sustainable development from 2015 to 2023?

RQ3. What are the research themes in the literature review and the areas for future research?

## 2. Materials and Methods

The PRISMA model was used in this research. The research obtained data from SCOPUS and Web of Science, which are widely used and reliable sources of scientific publications in social sciences. These sites are credited for broader coverage and citation count. Multiple software packages were used for data analysis, namely Bibliometric R package (4.3.2), VoS Viewer (1.6.20), Nvivo (14), and Excel. The Bibliometric R package and VoS Viewer were used for bibliometric analysis, Nvivo was used for thematic analysis of the top ten articles, and Excel for charts. The documents were uploaded in Nvivo, and five key steps were followed to develop the themes. The initial steps were data familiarization and coding to explore and gain an initial understanding of the data within NVivo and to create nodes by highlighting relevant text segments and categorizing them under specific themes, respectively. Subsequent steps were identifying themes by grouping related codes, reviewing and refining themes, and interpreting and reporting them.

The keywords “dairy sector”, “dairy industry” OR “milk production” OR “livestock farming”, “sustainability”, “sustainable development goals”, and “SDGs” occurring in the abstract, keywords, and titles were used to extract publications from both SCOPUS and Web of Science. They were limited to articles published from 2015 in English and relevant research fields.

The exact research keyword combination was as follows:

(TITLE-ABS-KEY ((“dairy sector” OR “dairy industry” OR “milk production” OR “livestock farming”)) AND TITLE-ABS-KEY ((“sustainability” OR “sustainable development goals” OR “SDGs”))) AND PUBYEAR > 2015 AND (LIMIT-TO (SRCTYPE, “j”)) AND (LIMIT-TO (DOCTYPE, “ar”)) AND (LIMIT-TO (LANGUAGE, “English”)) AND (EXCLUDE (PUBYEAR, 2024)) AND (LIMIT-TO (SUBJAREA, “AGRI”) OR LIMIT-TO (SUBJAREA, “VETE”) OR LIMIT-TO (SUBJAREA, “SOCI”) OR LIMIT-TO (SUBJAREA, “BUSI”) OR LIMIT-TO (SUBJAREA, “ENVI”)) AND (LIMIT-TO (EXACTKEYWORD, “Sustainability”) OR LIMIT-TO (EXACTKEYWORD, “Cattle”) OR LIMIT-TO (EXACTKEYWORD, “Milk Production”) OR LIMIT-TO (EXACTKEYWORD, “Dairy Farming”) OR LIMIT-TO (EXACTKEYWORD, “Milk”) OR LIMIT-TO (EXACTKEYWORD, “Sustainable Development”) OR LIMIT-TO (EXACTKEYWORD, “Dairying”) OR LIMIT-TO (EXACTKEYWORD, “Dairy Industry”) OR LIMIT-TO (EXACTKEYWORD, “Dairy Cattle”) OR LIMIT-TO (EXACTKEYWORD, “Milk Yield”) OR LIMIT-TO (EXACTKEYWORD, “Dairy”) OR LIMIT-TO (EXACTKEYWORD, “Dairies”) OR LIMIT-TO (EXACTKEYWORD, “Farming System”) OR LIMIT-TO (EXACTKEYWORD, “Grazing Management”)).

**Figure 1:**
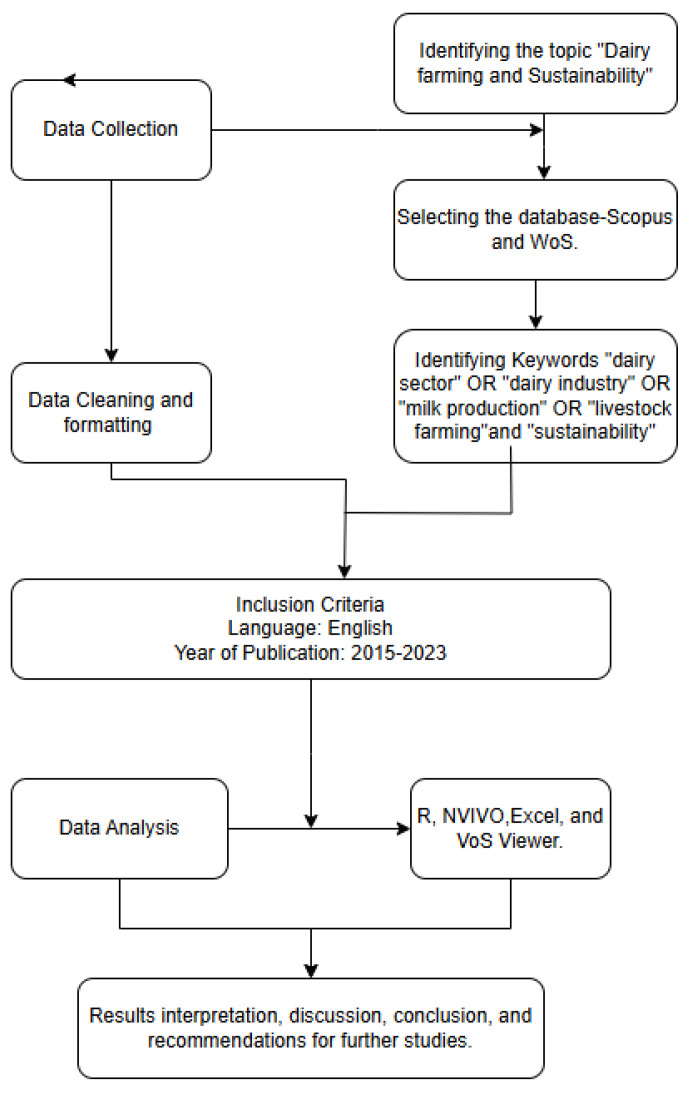
Search methodology.

The search methodology is illustrated in Figure 1.

The results obtained from the above search methodology are shown in Table 1. These search results are from SCOPUS and Web of Science (WoS).

The third section shows the research findings based on the three research questions. Each question was addressed independently.

## 3. Results

The research findings are divided into three parts based on the research questions. The first section presents the publication trends, the second section presents the most prolific scholars, articles, journals, and countries contributing to dairy farming and sustainable development, and third section presents the research themes from the literature review and areas for future research.

### 3.1. The Publication Trends

This section explores the publication trends in dairy farming and sustainability from 2015. It shows the annual number of publications from 2015 to 2023, the articles’ annual growth, and the price law. Each of these measures is explained independently.

#### 3.1.1. Annual Number of Publications from 2015 to 2023

**Figure 2:**
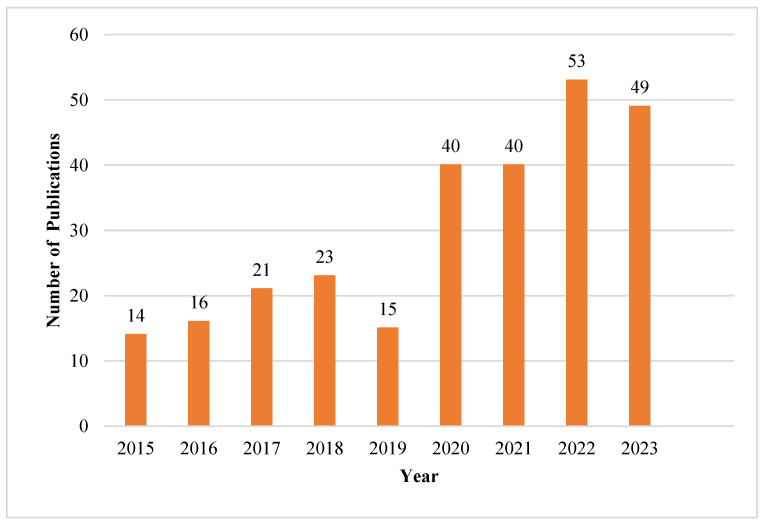
Annual publications from 2015 to 2023.

We examined the number of scientific publications published from 2015 to 2023 to identify the trends, and the results are shown in Figure 2.

**Figure 3:**
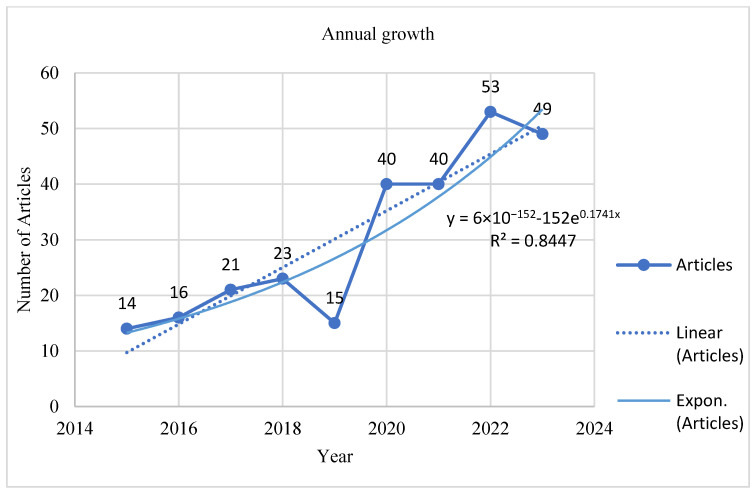
Articles’ annual growth over time.

Researchers’ interest has been drawn to the field, as illustrated in Figure 2, which shows a constant increase in publications on dairy farming and sustainability from 2015. Nonetheless, there was a decline in 2019, followed by a more than twofold increase in the following years. This can be attributed to the global focus on COVID-19 and the closure of most research institutions and universities. In addition, most researchers focused on the impact of COVID-19. In 2023, there was a slight decline in publications compared to 2022. This trend is further supported by the price law, which is illustrated in Figure 3.

#### 3.1.2. Price Law

Price law shows productivity distribution in a scientific discipline. It states that only a few scholars make significant contributions and discoveries in a particular discipline [30]. This law helps to identify key contributors in a research area and productivity over time. We created an exponential growth curve to estimate productivity over time, and the results are shown in Figure 3. The results show an exponential growth value of 0.1741 over time. The model has a strong goodness of fit, explaining 84% of the data variability. These findings support the prior high productivity assertion. The increase in scientific research on dairy farming and sustainability can be explained by the adoption of sustainable goals in 2015. Since then, countries have formulated sustainability-based policies that govern their economic operations and production. Based on this law, the increase in publications could be driven by the expansion of academic journals and institutional pressures to publish research on topics other than groundbreaking discoveries.

### 3.2. Prolific Sources, Articles, and Authors

This section shows the most relevant sources, most cited journals, the source impact, most cited articles, prolific scholars, and countries’ production over time.

- Most Relevant Sources

**Figure 4:**
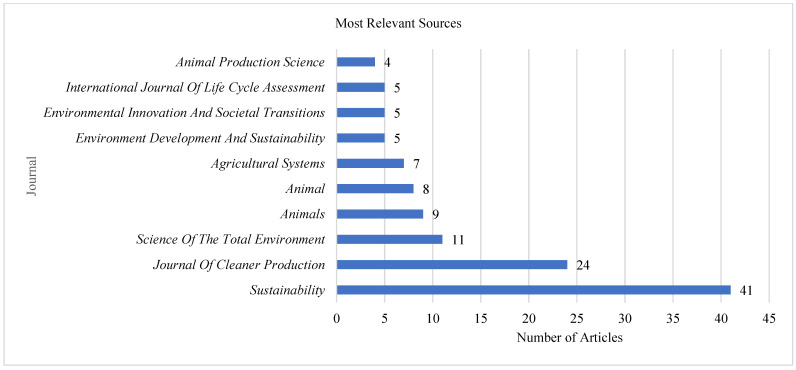
Top ten most relevant journals.

We examined the most relevant sources in dairy farming and sustainability, and Figure 4 shows the top ten journals.

The *Sustainability* journal has the most articles, followed by the *Journal of Cleaner Production* and the *Science of the Total Environment* Journal. Most journals had five publications. *Animal Production Science* has the least publications (4). Most of the journals, specifically four of them, are in the agricultural field since dairy farming is majorly covered in agriculture. Nonetheless, environment, sustainability, and production journals have also explored the field. This implies that sustainable dairy production is a multidisciplinary area that has drawn attention from scholars in diverse areas.

- Most Cited Journals

**Figure 5:**
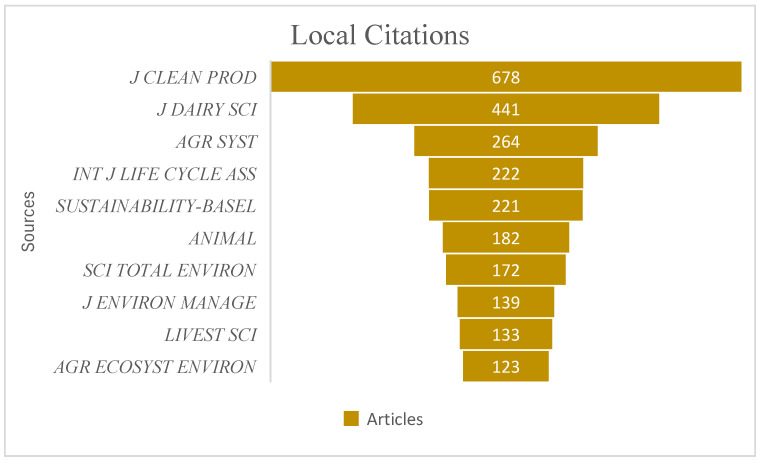
Most cited journals.

We further examined the local citations of the top ten listed journals to identify the journal with the most citations. The results are shown in Figure 5.

The *Journal of Cleaner Production* has the highest local citations, 78, followed by the *Journal of Dairy Science* with 441 citations, and *Agriculture Systems* (264). The journals had at least 123 citations. The journal of *Agriculture, Ecosystems, and Environment* had the least citations. Multiple journals, such as *Sustainability*, *Animals*, and *Animal Production*, had the most articles, as shown in the previous analysis, but do not appear among the top ten. This implies that the most cited journals may have fewer but highly cited articles in dairy farming and sustainability.

- The Sources’ Impact

Further, we examined the journals’ H-index values to determine their impact. The H-index measures a researcher’s scientific output, and Hirsch (2005) [31], explained that “A scientist has index H if H of his or her Np papers have at least H citations each and the other (Np-h) papers have less or equal H citations each”. It is expressed as follows:

H = max {h: at least h papers have h or more citations each}

**Figure 6:**
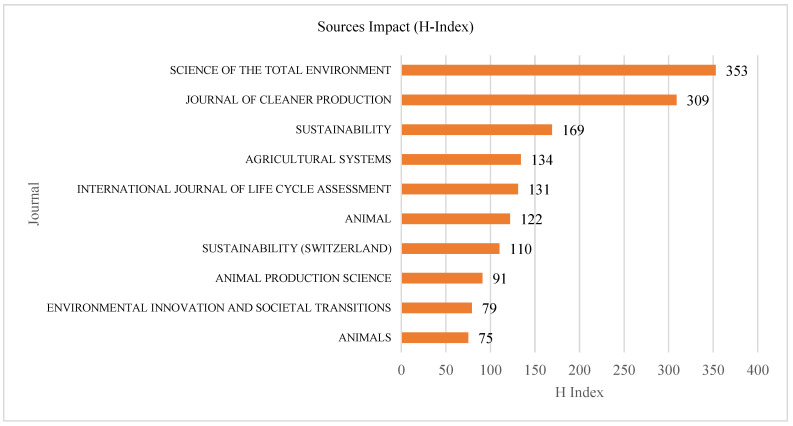
Sources’ impact (H-index).

This index is highly preferred because it combines impact and quantity and measures scientific output objectively. The results show that of the top ten sources, the smallest H-index was 3, and the highest was 14 as shown in Figure 6. The *Journal of Cleaner Production* had the highest H-index (14), while *Animal Production Science* had the smallest (3). Most journals had an index of 5. Journals with high citation scores have a high H-index, and most of the journals in the top citation list have a high H-index. This implies that journals on sustainability have a higher ranking due to increased research interest in sustainability.

- Most Cited articles

**Figure 7:**
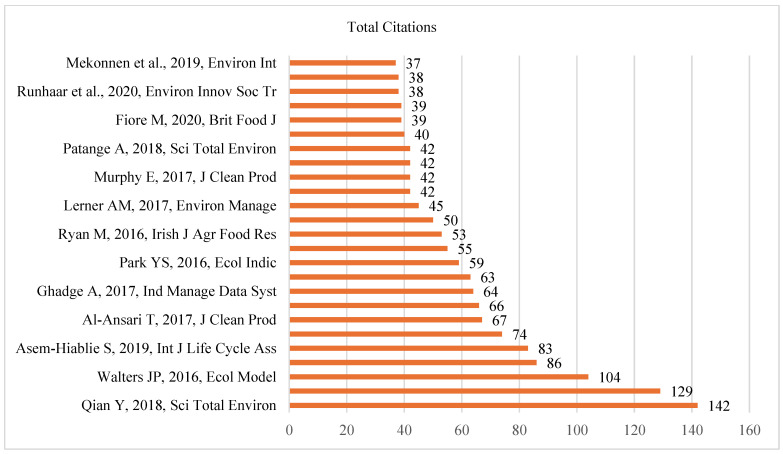
Most cited articles [32,33,34,35,36,37,38,39,40,41,42,43,44].

We further explored the top ten most cited articles to identify influential studies, explore research trends, and measure the research impact. Figure 7 shows the top most cited articles globally in dairy farming and sustainability.

Qian’s article (2018) [32] is the most cited article, with 142 citations, followed by those by Walters (2016) [33] with 129 and Asem (2019) [34] with 104 citations. The least cited article in the list has 37 citations. Nonetheless, articles with the most citations were published earlier compared to others. Additionally, there are multiple newer publications with higher citations. For instance, Asem (2019) [34] is cited more often than Murphy (2017) [40] and Park (2016) [37]. Therefore, though articles published earlier have a citation upper hand, a recent published study with strong relevance can attract more citations as well.

- **Journal Listings and Article distribution Based on Bradford Law**

The Bradford law is a widely used bibliometric law that examines the output of scientific journals. The law states that “if scientific journals are arranged in order of decreasing productivity of articles on a given subject, they may be divided into a nucleus of periodicals more particularly devoted to the subject and several groups or zones containing the same number of articles as the nucleus”. Table 2 shows a summary of journal listings and article distribution based on the Bradford law.

The results show that zone 1 had a minimum of five journals, which increased by sixfold in zone 2. In addition, articles in zone 2 increased by threefold in zone 3. The articles are uniformly distributed in the three zones. Each zone has about 92 articles (33%). The results align with Bradford’s law, which requires articles to be uniformly distributed across the zones, and a few journals should contain most articles.

- Prolific Scholars

**Figure 8:**
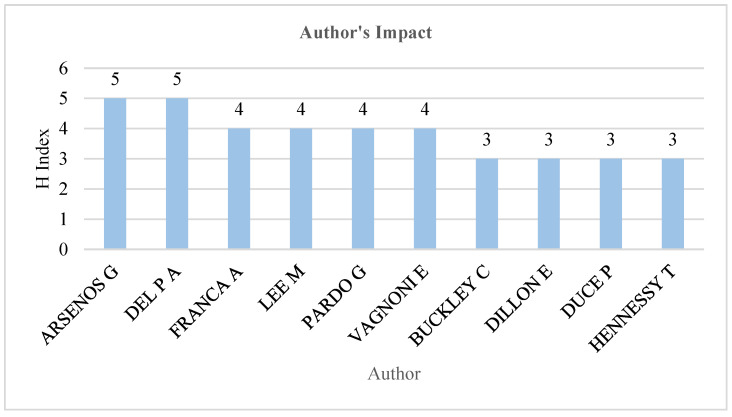
Authors’ impact (H-index).

This study examined the most prolific scholar based on the H-index. Similarly to journal impact, the H-index is used to measure the impact of scholars in a given field. Figure 8 shows the top ten scholars in dairy farming and sustainability.

Arsenos G. and Del P. are the most prolific scholars with five publications authored individually or co-authored with other scholars. Franca A., Lee M., Pardo G., and Vagnoni E. are the second most prolific scholars in dairy farming and sustainability since 2015, with four publications authored individually or co-authored with other scholars. Buckley C., Dillon E., Duce P., and Hennessy T. have three individual or co-authored publications.

- Number of Articles Published by Country Over Time

**Figure 9:**
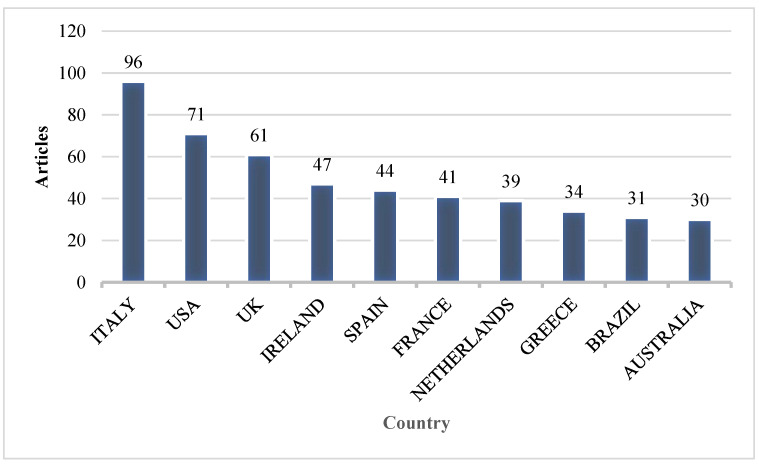
Number of articles published by country over time.

We examined the top ten countries with the most articles to identify the country leading in studies related to dairy farming and sustainability. The findings are shown in Figure 9.

The results show that Italy is leading with 96 publications, followed by USA (71), United Kingdom (61), Ireland (47), Spain (44), France (41), the Netherlands (39), Greece (34), Brazil (31), and Australia (30). Italy and Australia are the leading milk producers in the world, which can be attributed to their interest in researching sustainable practices in dairy farming. It is worth noting that no Asian or African country is featured among the top ten.

- Lotka’s Law

Lotka’s law is used to evaluate the productivity of authors. The law states that “The number of authors making n contributions is 1/*n*^2^ of those making one, and the proportion of contributors making a single contribution is 60%”. Table 3 shows the productivity of authors based on Lotka’s law.

The results show that 87% of the researchers in dairy farming and sustainability had published one article, 10% published two articles, and 1.5% published three articles. The results do not align with the assumption of Lotka’s law, which requires 60% of the authors to have a single publication.

- Co-occurrence of Keywords

**Figure 10:**
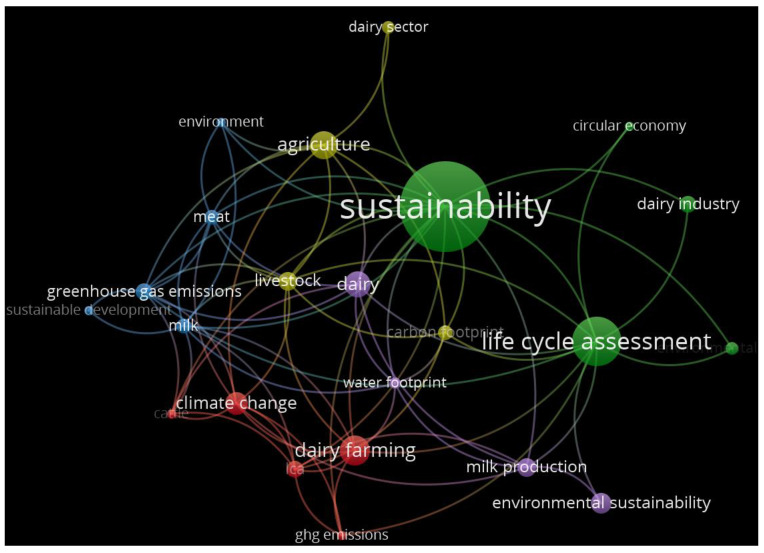
Co-occurrence of keywords.

We examined the co-occurrence of keywords to identify research trends, map knowledge domains, detect research fronts, and explore interdisciplinary connections in dairy farming and sustainability. Figure 10 below shows the keywords related to this study.

According to the output, cluster 1 contains sustainability as the keyword, followed by life cycle assessment. Other keywords in different clusters are dairy farming, climate change, environmental sustainability, agriculture, greenhouse emissions, milk production, circular economy, and water footprint. The co-occurrence of lifecycle assessment and sustainability emphasizes the examination of the environmental impact of dairy farming practices across the entire lifecycle of dairy products from production to consumption. It implies that researchers are examining approaches to make dairy farming operations sustainable by optimizing the use of limited resources and reducing environmental footprints. The occurrence of dairy farming in multiple clusters indicates its centrality to the overall research landscape as researchers examine various dairy farming aspects, such as production approaches, economic viability, and animal welfare. Moreover, the co-occurrence reflects a shift towards evaluating environmental impacts comprehensively. The presence of keywords like circular economy and water footprint suggests an increasing focus on resource efficiency and reducing emissions, highlighting the evolving priorities in dairy sustainability research, shifting from traditional production concerns to holistic environmental management approaches.

### 3.3. A Literature Review of the Top Twenty Most Cited Articles

We reviewed the top ten articles with the greatest impact to identify themes, trends, and opportunities for future research. Table 4 below shows the literature review of the top twenty studies.

A review of the literature, including the top 20 articles regarding the sustainability of the dairy sector, helped to identify the thematic issues associated with dairy farming and areas of further research. This section explores each theme and provides suggestions for future research.

### 3.4. Thematic Analysis

This section explores the themes identified from the literature review, namely livestock management, feed production and management, innovation, stakeholders’ involvement, logistics and supply chain management, and waste management.

- Theme 1. Livestock Management

The reviewed literature shows that the evolving landscape of environmental sustainability within the dairy sector is intricately tied to the management of livestock. Intensive dairy systems increase the amount of greenhouse gas emissions, thereby impairing conservation measures and posing a significant challenge to overall sustainability efforts [34,38]. Consequently, current dairy farming practices require a re-evaluation in favor of more sustainable approaches. In addition, the results imply that rotational fallowing allows for land rejuvenation and soil health to flourish, thus enhancing ecological sustainability [46]. The literature supports the pivotal role of the cattle production phase, spanning from feed production to cow–calf rearing and finishing, in sustainability [34]. This phase cuts across multiple environmental categories, which signifies the benefits of targeted conservation interventions within livestock management. Therefore, environmental sustainability in the dairy sector requires a comprehensive approach that prioritizes innovative strategies for livestock management; optimizing the cattle production phase and integrating rotational fallowing techniques can not only mitigate greenhouse gas emissions but also foster ecological resilience.

- Theme 2. Feed Production and Management

Feed production and management within the dairy sector have multiple complexities that should be addressed to enhance environmental sustainability. The key and notable challenge is the shift in dietary preferences [46]. The feed production process requires re-examining to meet consumer demands sustainably. Moreover, the animal feed supply chain is characterized by food wastage, thus creating resource inefficiencies and adding to environmental degradation [47]. The literature shows that irrigating cattle feed crops often results in water wastage, which presents a substantial concern for sustainable water management [48]. Besides irrigation, dairy food production approaches require optimization [34]. The reliance on grains in formulating feeds is associated with intensive agricultural practices that amplify environmental degradation and resource depletion [38]. Additionally, poor feeding strategies and the inept utilization of feed resources further aggravate environmental strain, demanding a shift towards more sustainable practices [54].

To address these complex challenges in the feed production and management value chain, it is crucial to adopt multifaceted approaches that promote environmental sustainability within the dairy sector. One key solution is adopting a market-driven transition to more sustainable feed production and management approaches that realign feed production and usage with shifting dietary preferences and SDGs [46]. Concurrently, systematic interventions, such as resource optimization and efficiency, should be implemented in each stage [48]. Secondly, optimizing feed production, embracing sustainable alternatives (green water irrigation), and developing new innovations in water management approaches and irrigation techniques can enhance resource efficiency, address water wastage, and reduce the environmental footprint of feed production [48]. Thirdly, strategic interventions, focusing on reducing reliance on grains in feed formulations and improving harvesting practices to enhance forage quality and quantity, are vital approaches towards sustainability [54]. In addition, feeding systems that are balanced and phased alongside innovative feed formulations and targeted mineral supplements are essential. Thirdly, it is central to align improved productivity with environmental imperatives. Ecologically sound practices increase crop and pasture yields while conserving the environment [34]. Finally, the literature underscores the importance of transitioning from intensive agriculture to organic farming [34]. This transformative approach integrates ecological and biological principles into agricultural food production. Stakeholders can navigate animal feed production complexities and foster a resilient and environmentally conscious industry by embracing these comprehensive solutions.

- Theme 3. Innovation

Innovation emerges as a central theme in the dairy sector that enhances environmental sustainability in the analyzed literature. It provides numerous transformative solutions that are poised to reshape industry practices in the quest to enhance sustainability. Foremost, the use of robots can help to optimize dairy farming operations [49]. They revolutionize efficiency while minimizing resource inputs and align with sustainability objectives. Automated milking systems and precision livestock management are examples. In Finland, an automated milking system has been adopted, which reduced the time and labor required for milking by 5.2 to 2.0 h per day while increasing milk yield [55]. Additionally, harnessing proper solar energy and reducing reliance on fossil fuels are sustainable energy solutions in the sector that can mitigate greenhouse gas emissions associated with traditional energy sources [37].

Moreover, innovative socio-institutional frameworks and incentives foster the adoption of sustainable agricultural practices among dairy farmers as they support and provide incentives for environmentally friendly approaches [51]. These frameworks catalyze a shift towards more sustainable production methods; for instance, downscaling ethical allocation principles to the individual level further reinforces this transition and empowers farmers to make informed decisions that prioritize sustainability within their operations [21,52].

Developing consumer-centric innovations also holds promise in driving demand for sustainable dairy products [42]. Innovations, such as eco-friendly packaging and traceability technologies, encourage consumers to make environmentally conscious choices and help to incentivize sustainable practices throughout the dairy supply chain [42].

Innovative feed production and feeding practices are central promoters of sustainability within the dairy sector as they optimize feed formulations, reduce waste, and incorporate alternative feed sources [54]. These practices enhance resource efficiency and minimize the environmental impact associated with feed production.

Conclusively, it is evident that innovation is a catalyst for achieving sustainability within dairy farming as it revolutionizes operational practices, promotes renewable energy sources, fosters institutional support, empowers consumers, and optimizes feed production. Embracing these innovative solutions can help stakeholders to navigate the complexities of dairy farming while simultaneously advancing environmental sustainability goals.

- Theme 4. Stakeholder Involvement

Stakeholders is the fourth theme present in the reviewed literature. It is evident that stakeholders play a central role in driving and pushing sustainability initiatives in the dairy sector [42]. However, the research highlights numerous stakeholder engagement challenges and opportunities that directly affect the dairy sector’s ability to attain sustainability goals.

A lack of consensus among stakeholders is a significant impediment to attaining the SDGs in the dairy sector identified in the literature [36]. The disagreement stems from divergent interests, conflicting priorities, and limited communication channels between stakeholders within the dairy value chain [50]. To address this challenge, fostering coordination between key players and government sectors, such as the environmental, agricultural, and education sectors, is imperative to facilitate knowledge sharing, policy alignment, and capacity-building initiatives [51]. Establishing common goals and promoting cross-sectoral dialog enable stakeholders to work together more effectively to overcome challenges and drive sustainable practices within the dairy sector.

In addition, cooperatives that bring together stakeholders are catalysts for innovation in dairy farming [42]. For instance, collaborative efforts between farmers, researchers, policymakers, and other stakeholders can spur the development and adoption of innovative practices and technologies as it helps to leverage collective expertise and resources, which facilitate knowledge transfer, experimentation, and the scaling of sustainable solutions [36]. This inclusive approach or cooperation between key players empowers stakeholders to co-create initiatives that address the sector’s unique challenges while advancing sustainability objectives.

The opportunity to adopt a Strategic Business Model (SBM), as supported in the literature, can facilitate continuous interaction with various stakeholders. Engaging with stakeholders allows dairy farmers and industry players to gain valuable insights, identify emerging trends, and anticipate future challenges [50]. In addition, actively involving stakeholders in strategic decision-making processes, such as developing business models and dairy farming enterprises, can help to align dairy farming operations with sustainability principles [51]. This collaborative approach enhances the effectiveness and long-term viability of the sector by fostering transparency, accountability, and shared ownership of sustainability initiatives.

In essence, engaging stakeholders drives sustainability efforts within the dairy sector [36]. Consequently, by addressing challenges such as the lack of consensus, fostering collaboration, and leveraging diverse expertise, stakeholders can collectively foster sustainability in the industry [50]. Dairy farming stakeholders can unlock innovation, resilience, and growth opportunities by adopting coordinated action and inclusive decision-making, which are essential to safeguard the environment and support local communities [39].

- Theme 5. Logistics and Supply Chain Management

Logistics and supply chain management is an additional theme identified in the reviewed literature that plays a critical role in both operational efficiency and environmental sustainability.

Greenhouse gas emissions and food wastage from logistics and transport within the dairy sector are common [47]. They stem from the transportation of dairy products, feed, and other inputs that often involve the extensive use of fossil fuels and food spoilage and wastage during transportation [35]. Often, the dairy sector is characterized by inefficient transportation and poor storage that adversely affect the environment and lead to wastage of feeds.

To address these challenges, it is imperative to adopt solutions that seek to improve logistics and supply chain management in dairy farming. Implementing real-time decision support systems provides timely insights and data-driven recommendations for logistics management that can enhance operational efficiency [49]. The dairy sector can leverage advanced analytics and technology to optimize transportation routes, minimize idle time, and reduce fuel consumption, thereby improving the overall efficiency of resources and mitigating the emission of greenhouse gases [37]. Furthermore, precision agriculture, automation, and data-driven decision-making can help farmers achieve higher output and use input more efficiently by optimizing resource allocation, minimizing waste, and maximizing productivity [37]. These approaches can help dairy farmers to reduce their environmental footprint while enhancing the profitability and resilience of the sector. In addition, the efficient management of the cold chain is essential to preserve the quality and safety of dairy products from the farm to the consumer [47]. By implementing proper storage, handling, and transportation practices, farmers in the dairy sector can minimize food wastage, reduce financial losses, and mitigate environmental impacts associated with spoilage and waste [47].

- Theme 6. Waste Management

Waste management is another theme highlighted in the literature as critical within the dairy sector. Poor handling of waste can result in significant greenhouse gas emissions and environmental degradation [35]. The most common wastes in dairy farming are solid wastes and wastewater effluents [35]. They pose challenges that require innovative solutions to mitigate their impact on the environment. Poor management of solid waste generated from dairy farming operations results in the release of greenhouse gases [35]. To avert this, firstly, carbon capture technology should be integrated with Biomass Integrated Gasification Combined Cycle (BIGCC) systems to effectively neutralize the gases emitted by solid waste [35]. This system not only captures but also stores carbon dioxide emissions, thus mitigating their environmental impact while producing useful energy. Additionally, recycling solid waste from the dairy sector into bioenergy offers a sustainable alternative to conventional waste disposal methods, which reduces both waste accumulation and constant reliance on fossil fuels [54]. Methane gas emitted from the dairy farming sector can be addressed by improving manure storage and introducing better handling methods [49]. The implementation of anaerobic digestion systems or composting techniques can help farmers to efficiently manage manure while capturing methane for energy production [49]. In addition to improving manure storage and handling approaches, wastewater can be safely decontaminated using Advanced Oxidation Processes (ACPs), which mitigate pollution in water bodies, thus safeguarding the environment and human health [41].

There are innovative biological approaches highlighted in multiple studies that can help to manage waste and enhance sustainability in the sector. Integrating algae, such as the production of Oedogonium, into waste management practices of intensive animal production presents an innovative approach to nutrient recovery [53]. Oedogonium utilizes nitrogen and phosphorous in waste streams to grow biomass that can be used in biofuel production and animal feed supplementation. This closed-loop system reduces pollution and enhances resource efficiency within the dairy farming ecosystem. Furthermore, better recycling of human food wastes and human-inedible food components into animal feed offers a sustainable solution to reduce waste and improve resource utilization as it diverts food waste from landfills and integrates it into the feed supply chain [54]. Thus, effective waste management practices are crucial for promoting sustainability within the dairy farming sector and implementing innovative solutions such as carbon capture technology, waste recycling, and nutrient recovery systems. Stakeholders can minimize greenhouse gas emissions, reduce pollution, and enhance resource efficiency, contributing to a more sustainable and resilient dairy farming industry.

## 4. Discussion, Conclusions, and Recommendations

Dairy farming is an important sector in achieving the SDGs, particularly those related to zero hunger, clean water and sanitation, climate action, and responsible consumption and production. The bibliometric analysis of sustainability in the dairy sector shows a rise in scholarly interest in dairy farming and sustainability, as evidenced by an increase in publications since the adoption of the SDGs in 2015. However, the variations in research activities over time, for instance, a decline in 2019, followed by rapid growth in the following years, points to underlying dynamics that require further studies. The decline in studies in 2019 can be attributed to the outbreak of COVID-19. Nonetheless, more studies should be conducted to ascertain the cause.

The exponential growth curve analysis underscores the robustness of research output due to the significant rise in productivity over time and aligns with the adoption of the global sustainable goals in 2015. These findings are a clear indicator of the global commitment to addressing current environmental challenges and promoting sustainable development in dairy farming. Furthermore, the complexity of sustainability in the dairy sector and the multidisciplinary nature of dairy production is shown by a wide range of journals contributing to the area. Notwithstanding the disparities in journal visibility and impact, the review of articles with a high number of citations shows historical foundational research and current advances shaping dairy farming and sustainability research discourse.

From a thematic analysis, it is evident that achieving sustainability in dairy farming requires addressing complex challenges across the value chain. While dairy farming is integral to providing dairy products and food, the sector faces significant environmental concerns stemming from livestock management, feed production, waste management, stakeholders’ involvement, innovation practices, and supply chain management. According to the analysis, there is a need to shift towards sustainable livestock management practices, such as rotational grazing and optimized feed formulations, since intensive dairy systems substantially contribute to greenhouse gas emissions. Moreover, there is a need to reduce food wastage and adopt efficient feed utilization approaches. Innovation emerges as a key driver of sustainability and source of opportunities to strengthen sustainability and achieve SDGs. On the other hand, collaboration between stakeholders, such as farmers, policymakers, and consumers, is essential for developing and implementing sustainable solutions. Logistics and supply chain management practices strongly influence the environmental impact and underscore the need to strengthen efficiency and adopt real-time decision support systems in the sector. It is imperative to recognize that management activities and operations that require human involvement in the dairy sector are key impediments to the sustainability of dairy farming, and concerted efforts across the entire production and supply chain are needed. Future studies can examine funding trends and collaboration networks across institutions and countries and themes in developing countries vis-à-vis developed economies. In addition, researchers can examine the role of precision farming technologies or the integration of consumer behavior changes into sustainability models and explore the links between animal welfare and sustainable dairying.

## Figures and Tables

**Table 1:** The search results.

Search Results		
Terms that were searched are “dairy sector” OR “dairy industry” OR “milk production” OR “livestock farming” AND “sustainability” OR “sustainable development goals” OR “SDGs”		
	Scopus	WoS
Article	134	243
Conference	19	7
Paper Review	28	6
Book Chapter	14	6
Book	1	2
**Limit to document type, relevant area**		
Articles	134	243
Total	377	
Cumulative Total	102	
Duplicates	275	
Remaining	0	
Abstract Review	275	
Articles Reviewed		

**Table 2:** Summary of journal listings and article distribution.

Bradford Law					
	Journals			Articles	
	N	%		N	%
Zone 1	5	4%	Zone 1	93	34%
Zone 2	33	26%	Zone 2	91	33%
Zone 3	90	70%	Zone 3	90	33%
	128			274	

**Table 3:** Authors’ productivity.

Documents Written	No. of Authors	Proportion of Authors
1	1025	0.872
2	123	0.105
3	18	0.015
4	6	0.005
5	4	0.003

**Table 4:** Literature review of top twenty most cited articles.

Author(s), Year, and Journal	Title of Paper	Citations	Contribution to Dairy Sector and Sustainability
Qian et al., 2018	Environmental status of livestock and poultry sectors in China under current transformation stage [32]	142	The paper provides policy recommendations for sustainable practices, including regulatory control, proper manure treatment, and financial incentives to promote ecological agriculture in China. Overall, the research underscores the need for sustainable livestock and poultry production.
Uwizeye et al., 2020	Nitrogen emissions along global livestock supply chains [45]	129	The article suggests targeted interventions to reduce N pollution, such as improved fertilizer policies and integrated livestock feed production. Global initiatives are needed to address N emissions while ensuring food security.
Walters et al., 2016	Exploring agricultural production systems and their fundamental components with system dynamics modelling [33]	104	The researchers evaluated three distinct production systems: crops only, livestock only, and an integrated crops and livestock system. They analyzed the role of each driver in determining sustainability differences among these systems. The greatest potential for sustainability exists in the crops-only production system.
Ritchie et al., 2018	The impact of global dietary guidelines on climate change [46]	86	Dairy intake is responsible for variations in emissions. Effective decarbonization requires not only a shift in dietary preferences but also a reevaluation of the recommendations supporting this transition. Improvements in feed production and use present significant opportunities for reducing environmental impacts.
Asem-Hiablie et al., 2019	A life cycle assessment of the environmental impacts of a beef and dairy system in the USA [34]	83	The study examines the environmental impact of livestock keeping. According to the research, identifying areas for improvement in feed production and use, such as increasing crop and pasture yields, could be key to reducing environmental impacts. The cattle production phase (feed and cow–calf phase), including feed production, cow–calf, and finishing, has the most influence on most environmental categories.
Mangla et al., 2019	Logistics and distribution challenges to managing operations for corporate sustainability: Study on leading Indian diary organizations [47]	74	The research highlights the environmental impact of the dairy industry, including high greenhouse gas emissions and food wastage. It suggests that addressing cold chain management is crucial for reducing wastage, financial losses, and environmental issues.
Al-Ansari et al., 2017	Integration of greenhouse gas control technologies within the energy, water, and food nexus to enhance the environmental performance of food production systems [35]	67	The paper introduces a system that recycles solid waste from the dairy sector into useful energy, specifically mentioning dairy manure as a feedstock for the gasification process. Carbon capture technology is integrated with BIGCC, transforming it into a negative greenhouse gas emission technology, which could result in a near carbon-neutral food system.
Richter et al., 2020.	Water scarcity and fish imperilment driven by beef production [48]	66	The research explores water scarcity and fish imperilment in cattle farming. According to the findings, irrigation of cattle feed crops is useful, and beef and dairy consumption is the largest consumer of river water in the Western United States, significantly impacting water scarcity and causing ecological imbalance. The study suggests that reducing cattle feed crops and rotational fallowing can enhance ecological sustainability.
Ghadge et al., 2017.	Implementing environmental practices within the Greek dairy supply chain: Drivers and barriers for SMEs [36]	64	The study established that internal as well as external drivers influence the implementation of sustainable practices in the FSC. However, internal drivers carry higher weightage in the drive towards sustainability.
Chen et al., 2017.	Social life cycle assessment of average Irish dairy farm [49]	63	The study noted that Irish dairy farming has positive social impacts on value chain actors and society, with predominantly positive impacts on the local community and generally positive impacts on workers. Improving manure storage, introducing better handling methods, and using real-time decision support for operational management, as well as robots, have the potential to reduce emissions that cause adverse “health and safe living condition” impacts on the local community.
Park et al., 2016	Energy and end-point impact assessment of agricultural and food production in the United States: A supply chain-linked Ecologically based Life Cycle Assessment [37]	59	Grain farming, dairy food, and animal production-related sectors were found to have the greatest shares in both environmental and ecological impact categories as well as endpoint impact. Integrating biological and ecological concepts into agri-food production will minimize the use of nonrenewable resources. Reducing food waste, improving the efficiency of operations and processes, using proper solar energy in the agri-food sector, and converting intensive agriculture into organic farming can enhance sustainability.
Ghadge et al., 2021.	Sustainability implementation challenges in food supply chains: a case of UK artisan cheese producers [50]	55	The analysis identified several key barriers, including the initial investment cost, firm size, and unawareness of government regulations. Internal barriers significantly dominate the implementation of sustainability practices in comparison to external barriers. A lack of consensus regarding the concept of sustainability by different stakeholders is observed to be an issue negatively affecting the level of integration in SMEs.
Ryan et al., 2016.	Developing farm-level sustainability indicators for Ireland using the Teagasc National Farm Survey [38]	53	The analysis undertaken in this study showed that dairy farms, followed by tillage farms, tend to be the most economically sustainable of the four farm systems examined. Intensive dairy systems produce more GHGs than other less-intensive systems, and the consistent pattern across all farm systems is the positive correlation between economic performance and environmental sustainability, driven by higher output and more efficient use of inputs. Increases in efficiency and productivity generate increased profits without increasing negative environmental consequences.
Lerner et al., 2017	Sustainable Cattle Ranching in Practice: Moving from Theory to Planning in Colombia’s Livestock Sector [39]	45	The article reviews the concepts and discussions associated with reconciling cattle production and conservation. It includes disaggregating the cattle sector regionally and by production type, defining the most important areas for conservation, and understanding geographical biophysical limitations and opportunities for production strategies. It also requires coordinating across government sectors, including environmental sectors, agricultural sectors, and education= and capacity-building sectors. TEfforts to encourage more sustainable cattle production strategies can be linked to international financial mechanisms and to national goals for restoration and carbon mitigation.
Vermunt et al., 2020.	Sustainability transitions in the agri-food sector: How ecology affects transition dynamics [51]	42	Agency-incentivizing change among farmers can enhance biodiversity-friendly practices. Novelty institutional change consists of new socio-institutional frameworks and incentives for farmers to change agricultural practices, often in the form of business models. Models for change cannot easily be scaled, replicated, or standardized.
Murphy et al., 2017	Water foot printing of dairy farming in Ireland [40]	42	The objective of this study was to determine the primary contributors to freshwater consumption in Irish dairy farms. The utilization of green water available at a low opportunity cost compared to blue water to produce milk demonstrates the sustainability of milk production in Ireland with respect to water consumption.
Hjalsted et al., 2021.	Sharing the safe operating space: Exploring ethical allocation principles to operationalize the planetary boundaries and assess absolute sustainability at individual and industrial sector levels [52]	42	The study provides a demonstration of the feasibility of the method by implementing the framework into two of the planetary boundaries (climate change and biogeochemical flows) for the dairy sectors in India, Denmark, and around the globe. A two-step process is proposed, first downscaling to the individual level using ethically founded allocation principles and then upscaling to any higher level than the individual (such as product, industry sector, or nation) through separate upscaling methods. This allows stakeholders to transparently assess their absolute sustainability status.
Patange et al., 2018	Assessment of the disinfection capacity and eco-toxicological impact of atmospheric cold plasma for treatment of food industry effluents [41]	42	This study also investigated the eco-toxicological impact of cold plasma treatment of the effluents using a range of aquatic bioassays. Continuous ACP treatment was applied to synthetic dairy and meat effluents. The study showed the proof of principle on the safe treatment of food sector wastewater effluents using ACP for decontamination, with useful efficacy within short periods of both treatment and retention times. ACP treatment was shown as a promising technology for the reduction and complete inactivation of key indicator microorganisms in model dairy and meat wastewater effluent.
Cole et al., 2015.	Bio recovery of nutrient waste as protein in freshwater macroalgae [53]	40	The aims of this study were to investigate the relationship between nitrogen supply, biomass productivity, and the quantity and quality of protein in the freshwater macroalga, Oedogonium. Integrating the production of Oedogonium into the waste management of intensive animal production will provide a mechanism to recover nutrients which, firstly, delivers a novel source of protein for the agricultural sector and, secondly, contributes to the environmental sustainability of intensive animal production through bioremediation.
Fiore et al., 2020	Stakeholders’ involvement in establishing sustainable business models: The case of Polish dairy cooperatives [42]	39	The aim of the study was to define a sustainable business model of dairy cooperatives and explore how stakeholders can contribute to innovation processes generated in this ecosystem. The findings of this paper show how the involvement of various stakeholders through cooperatives contributes to the development of innovations that meet customer expectations, thereby contributing to the creation of social, environmental, and economic values. The opportunity to adopt an SBM is the result of continuous interaction with various stakeholders who contribute in varying degrees to the sustainability of processes and value co-creation.
Makkar, 2016	Smart livestock feeding strategies for harvesting triple gain–the desired outcomes in planet, people, and profit dimensions: a developing country perspective [54]	39	The analysis and synthesis presented revealed that the efficient utilization of feed resources and application of appropriate feeding strategies are vital for the sustainability of the livestock sector. The author identified and explored a series of promising innovations and practices in feed production and feeding, including balanced and phased feeding; increases in the quality and level of use of forages in diets; reductions in the use of grains; harvesting forages when nutrient availability per unit of land is at the maximum level; targeted mineral feeding; reductions in feed losses; the use of straw-based densified feed blocks; better recycling of human food wastes and human-inedible food components to feed; new business models for the production and use of urea-ammoniated straws, urea–molasses blocks, forages, and silages in smallholder farms; and the use of underutilized locally available feed crops linked with the strengthening of seed development and distribution of infrastructure.

## Data Availability

No new data were created or analyzed in this study. Data sharing is not applicable to this article. For further enquiries, contact Farkasné Fekete Mária Magdolna at farkasne.fekete.maria@uni-mate.hu.
